# Dual-Wavelength Imaging of Tumor Progression by Activatable and Targeting Near-Infrared Fluorescent Probes in a Bioluminescent Breast Cancer Model

**DOI:** 10.1371/journal.pone.0031875

**Published:** 2012-02-13

**Authors:** Bang-Wen Xie, Isabel M. Mol, Stijn Keereweer, Ermond R. van Beek, Ivo Que, Thomas J. A. Snoeks, Alan Chan, Eric L. Kaijzel, Clemens W. G. M. Löwik

**Affiliations:** 1 Department of Endocrinology and Metabolic Diseases, Leiden University Medical Center, Leiden, The Netherlands; 2 Department of Otorhinolaryngology Head and Neck Surgery, Erasmus Medical Center, Rotterdam, The Netherlands; 3 Department of Tissue Regeneration, Percuros B.V., University of Twente, Enschede, The Netherlands; Stanford University, United States of America

## Abstract

Bioluminescence imaging (BLI) has shown its appeal as a sensitive technique for *in vivo* whole body optical imaging. However, the development of injectable tumor-specific near-infrared fluorescent (NIRF) probes makes fluorescence imaging (FLI) a promising alternative to BLI in situations where BLI cannot be used or is unwanted (e.g., spontaneous transgenic tumor models, or syngeneic mice to study immune effects).

In this study, we addressed the questions whether it is possible to detect tumor progression using FLI with appropriate sensitivity and how FLI correlates with BLI measurements. In addition, we explored the possibility to simultaneously detect multiple tumor characteristics by dual-wavelength FLI (∼700 and ∼800 nm) in combination with spectral unmixing. Using a luciferase-expressing 4T1-luc2 mouse breast cancer model and combinations of activatable and targeting NIRF probes, we showed that the activatable NIRF probes (ProSense680 and MMPSense680) and the targeting NIRF probes (IRDye 800CW 2-DG and IRDye 800CW EGF) were either activated by or bound to 4T1-luc2 cells. *In vivo*, we implanted 4T1-luc2 cells orthotopically in nude mice and were able to follow tumor progression longitudinally both by BLI and dual-wavelength FLI. We were able to reveal different probe signals within the tumor, which co-localized with immuno-staining. Moreover, we observed a linear correlation between the internal BLI signals and the FLI signals obtained from the NIRF probes. Finally, we could detect pulmonary metastases both by BLI and FLI and confirmed their presence histologically.

Taken together, these data suggest that dual-wavelength FLI is a feasible approach to simultaneously detect different features of one tumor and to follow tumor progression with appropriate specificity and sensitivity. This study may open up new perspectives for the detection of tumors and metastases in various experimental models and could also have clinical applications, such as image-guided surgery.

## Introduction

Both bioluminescence imaging (BLI) and fluorescence imaging (FLI) are widely used optical modalities for non-invasive detection of tumor progression in small animals. BLI is appealing for *in vivo* whole body imaging because of its exceptional sensitivity and almost negligible bioluminescent background [1], [2], [3]. However, under certain circumstances, BLI is not applicable. For example, BLI cannot be employed in tumor models lacking expression of bioluminescent reporters, such as in spontaneous- or chemical-induced cancer models. Moreover, the introduction of a foreign reporter protein, such as luciferase, may induce an immune response directed against the reporter protein itself in syngeneic mouse models of cancer [4], [5], [6], [7], [8]. Finally, for clinical applications, such as image-guided surgery [9], BLI is also not applicable as it requires genetic modification of the targeted cells.

Recent advances in the development of tumor-specific injectable near-infrared fluorescent (NIRF) probes make FLI a promising alternative to BLI. The use of NIRF probes has several advantages. For example, in the near-infrared (NIR) region (600–900 nm), the absorption coefficient of major light absorbers in tissues (e.g., hemoglobin and water) is minimal, which improves the photon penetration depth. Moreover, the autofluorescence of tissues in the NIR region is minimal, which provides a high contrast between target and background fluorescence [10], [11], [12]. Currently, there are two main types of commercially available NIRF probes. Firstly, the so-called protease-activatable probes which can visualize tumors via activation by enzymes, such as matrix metalloproteinases (MMPs) or cathepsins, which are over expressed by various tumors and their surrounding stroma [13], [14], [15], [16], [17], [18]. Secondly, targeting probes which can recognize tumor tissues by binding to specific membrane targets, like glucose transporters (GLUTs) or epidermal growth factor receptors (e.g., EGFR, HER2) [19], [20], [21], [22]. These transporters and cell-surface receptors are over expressed in many different tumor cells because of their elevated glycolysis and proliferation [23], [24], [25], [26], [27]. These enzyme activatable and targeting probes were labeled with a 700 nm or 800 nm fluorophore, respectively, enabling their visualization simultaneously using dual-wavelength imaging. This method, with the introduction of spectral unmixing, extends the number of measurements made in the same animal and offers more accurate biologic observations *in vivo* [28].

The aim of the present study was to explore the use of FLI as a viable and sensitive alternative to BLI. We also investigated the possibility to simultaneously detect multiple tumor characteristics by using FLI at two wavelengths in combination with spectral unmixing. For this, we employed different combinations of activatable and targeting NIRF probes in a luciferase-expressing 4T1-luc2 mouse breast cancer model and assessed the correlation between FLI and BLI measurements.

## Materials and Methods

### Ethics statement

All animal experiments were approved for animal health, ethics, and research by the Animal Welfare Committee of Leiden University Medical Center, the Netherlands (Approval DEC number 09050). All animals were five week-old BALB/c nu/nu female mice (Charles River Laboratories, France) and received humane care and maintenance in compliance with the “Code of Practice Use of Laboratory Animals in Cancer Research” (Inspectie W&V, July 1999).

### Cell culture

The mouse mammary gland cancer cell line, 4T1-luc2, expressing a codon-optimized luciferase gene (*luc2*) was obtained from Caliper Life Sciences (Hopkinton, MA). This cell line has been shown to spontaneously produce highly metastatic tumors which can metastasize to the lung, liver and lymph nodes while the primary tumor is growing in situ [2], [29], [30]. The cells were maintained in complete RPMI-1640 Medium (Gibco, Invitrogen, Carlsbad, CA).

### *In vitro* cell-based fluorescent assays

The specificities and sensitivities of ProSense680, MMPSense680 (Perkin Elmer Inc., Boston, MA), IRDye 800CW 2-DG and IRDye 800CW EGF (LI-COR Biosciences, Lincoln, NE) to detect 4T1-luc2 cells were assessed in cell-based assays. For dose-dependent experiments, 1×10^4^ cells were seeded in 96-well plates and maintained in RPMI-1640 medium at 37°C, in a humidified incubator containing 5% CO_2_. After overnight adhesion, probes with different concentrations were added to the cell culture: 0 to 45nM for ProSense680 and MMPSense680, and 0 to 10 µM for IRDye 800CW 2-DG and IRDye 800CW EGF. The cells were incubated with the activatable probes (ProSense680 and MMPSense680) for 24 hours. Cells were washed twice with phosphate buffered saline (PBS) before imaging, to remove unbound dye. For the targeting probes (IRDye 800CW 2-DG and IRDye 800CW EGF), cells were starved for two hours in serum-free low-glucose DMEM (Gibco, Invitrogen, Carlsbad, CA) to elevate GLUT-1 or EGFR expression levels. Subsequently, the IRDye 800CW 2-DG was added and the cells were incubated for an additional two hours. For the IRDye 800CW EGF, the cells were incubated with the probe for 15 minutes. Both assays were then stopped and fixed for 20 minutes with 4% formaldehyde. Cells were subsequently washed twice with PBS to remove unbound probes. For cell amount-dependent experiment, 4T1-luc2 cells were seeded with a serial of cell dilutions, from 2×10^4^ to 39 cells/well. After overnight adhesion, cells were incubated either with 22.5nM Prosense680 or with 100nM IRDye 800CW EGF, as described previously. The 96-well plates were scanned with the Odyssey Infrared Imaging System (LI-COR Biosciences, Lincoln, NE), at the 700nm and 800nm channels.

### Microscopic analysis

4T1-luc2 cells were cultivated and incubated with probes following the procedure described previously. At the end of probe incubation, the growth medium was discarded and the cells were washed with pre-warmed fresh medium. The carbocyanine dye DiI, and Hoechst 33342 (Invitrogen, Carlsbad, CA) were then added to stain the cell membrane and nucleus, respectively, according to the manufacturer protocol. Samples with ProSense680 or MMPSense680 were imaged with a confocal TCS SP5 microscope (Leica, Mannheim, Germany) using appropriate laser power. Samples with IRDye 800CW 2-DG or IRDye 800CW EGF were imaged with the Nuance multispectral imaging system (Cri, Inc., Woburn, MA) using a Xenon 75W lamp followed by spectral unmixing [31].

### *In vivo* optical imaging

To follow tumor growth longitudinally, 2×10^4^ 4T1-luc2 cells were implanted into the lateral thoracic mammary fat pad (MFP) of the nude mice. For the tumor cell detection limit experiment, different numbers of 4T1-luc2 cells (1×10^5^, 5×10^4^, 2.5×10^4^ and 1.25×10^4^) were implanted into four upper thoracic MFPs. The combination of Prosense680 and IRDye 800CW EGF was injected the day before measurement (day 0 and day 3). Throughout tumor inoculation and imaging procedures, the animals were anesthetized with isoflurane. For *in vivo* BLI, 150mg/kg of D-luciferin solution (SynChem, Inc., Elk Grove Village, IL) in PBS in a total volume of 50 µL was injected intraperitoneally 10 minutes prior to imaging. The animals were imaged with the IVIS 100 imaging system (Caliper Life Sciences, Hopkinton, MA). Regions of interest (ROI) from displayed images were selected to cover the tumor regions and quantified with Living Image software from Caliper Life Sciences, using protocols as described previously [32].

**Figure 1:**
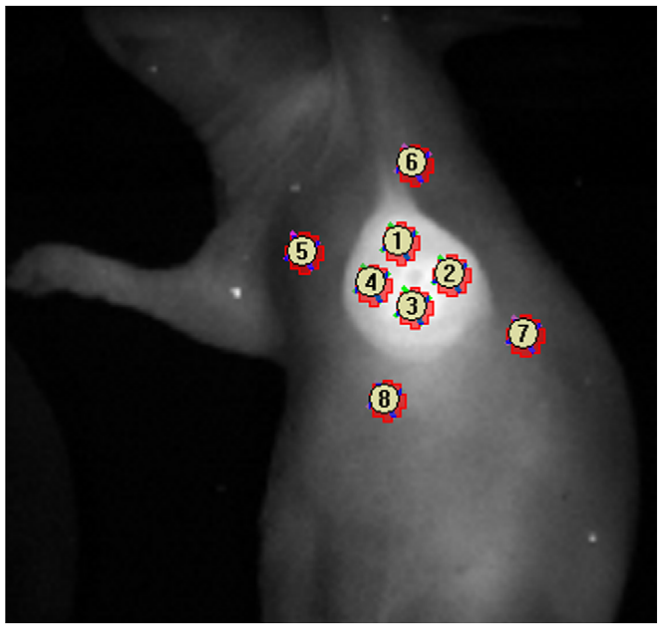
Schematic representation of the localization of the signal intensity areas used to calculate TBRs. TBRs were calculated by dividing the mean fluorescence intensity of four areas, of the same size, located inside the tumor region (1–4) by the mean fluorescence intensity of four areas located approximately 5 mm outside the tumor region (background fluorescence) (5–8).

For FLI, images were taken with the Maestro imaging system (Cri, Inc., Woburn, MA), 24-hour after probe injection of either the combination of ProSense680 (1.33nmol/100 µL in PBS) and IRDye 800CW 2-DG (10nmol/100 µL in PBS), or the combination of MMPSense680 (1.33nmol/100 µL in PBS) and IRDye 800CW EGF (1.33nmol/100 µL in PBS). The probes were injected intravenously in the tail vein. Each mouse was imaged together with a control mouse without probe injection in order to perform spectral unmixing [31]. After spectral unmixing, ROI from displayed images were selected as described before. The tumor-to-background ratio (TBR) was calculated by dividing the mean fluorescence values of the tumor region by the mean fluorescence values of the tumor border areas (Fig. 1).

For *ex vivo* BLI, 150mg/kg D-luciferin solution was injected into the mice five minutes prior to necropsy. Tissues of interest were excised and re-imaged for BLI and FLI signals. After imaging, excised tissues were subsequently prepared for standard histopathology analysis.

### Histological analysis

Primary tumors and possible metastatic tissues (lungs) were surgically removed and processed, either for paraffin-embedding or for cryosectioning. 8 µm slices of excised tumor tissues were scanned on the Odyssey machine using the 700nm and 800nm channels. The tumor sections containing ProSense680 and IRDye 800CW 2-DG were processed for CathpsinB (Calbiochem, Darmstadt, Germany) and GLUT-1 (abcam, San Francisco, CA) antibody staining. Sections containing MMPSense680 and IRDye 800CW EGF were used for MMP-9 (Santa Cruz Biotechnology, Inc., Heidelberg, Germany) and EGFR (abcam, San Francisco, CA) immuno-staining, respectively. Finally, the presence of metastases in lungs was confirmed by a pathologist in hematoxylin and eosin (H&E) stained sections.

### Statistical analysis

In order to analyze the correlation of FLI signals obtained from the NIRF probes with BLI signals from tumor cells, Pearson's r test was applied for statistical analysis, as a descriptor of the degree of linear association.

## Results

### *In vitro* characterization of different activatable and targeting NIRF probes

**Figure 2:**
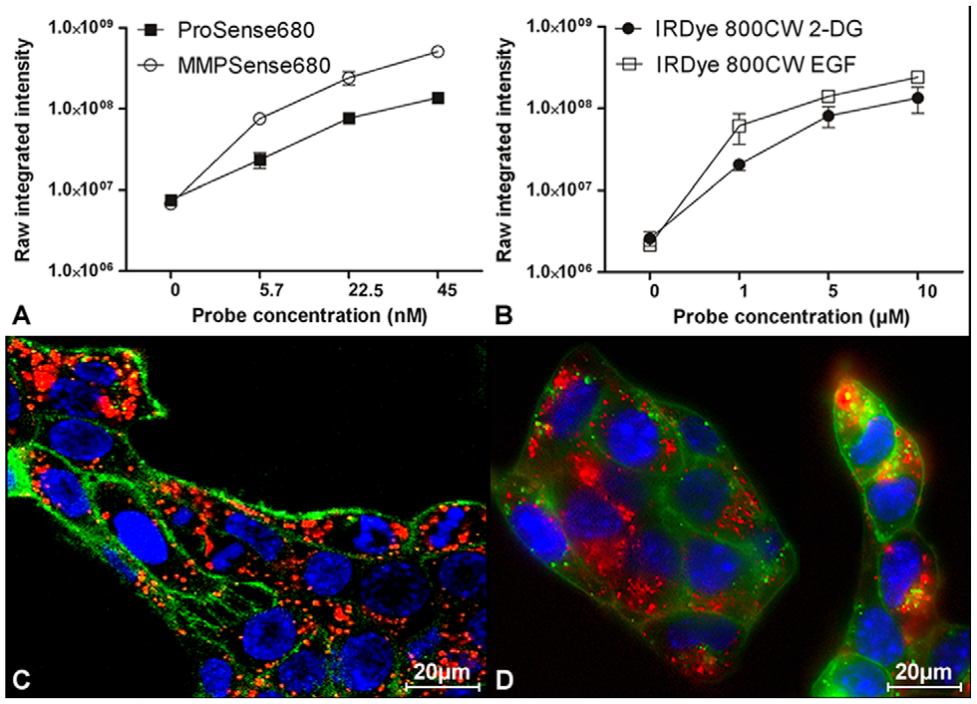
*In vitro* NIRF probe activation/binding assays. 4T1-luc2 breast cancer cells were seeded in 96-well plates and incubated with increasing concentrations of Prosense680 or MMPSense680 (A) and IRDye 800CW EGF or IRDye 800CW 2-DG (B) and were subsequently imaged. Intracellular accumulation of Prosense680 (45nM; 24 h incubation) was visualized by confocal microscopy (C). Intracellular accumulation of IRDye 800CW 2-DG (10 µM; 2 h incubation) was visualized by the Nuance multispectral imaging system (D). NIRF probes, cell nuclei and membranes are indicated in red, blue and green, respectively.

Activation of ProSense680 and MMPSense680 and binding of IRDye 800CW 2-DG and IRDye 800CW EGF by 4T1-luc2 cells were examined. Activation/binding assays, using a fixed initial number of cells incubated with different probe concentrations, were performed. A dose-dependent increase in the fluorescent signal intensity was found at increasing concentrations of each probe (Fig. 2A and B). As shown by microscopic analysis (Fig. 2C and D), the fluorescent signal (red) representing ProSense680 and IRDye 800CW 2-DG was found localized specifically in the cytosol of the cells. Similar results were found with MMPSense680 and IRDye 800CW EGF (data not shown).

### Sensitivity of FLI and BLI measurements *in vitro* and *in vivo*

**Figure 3:**
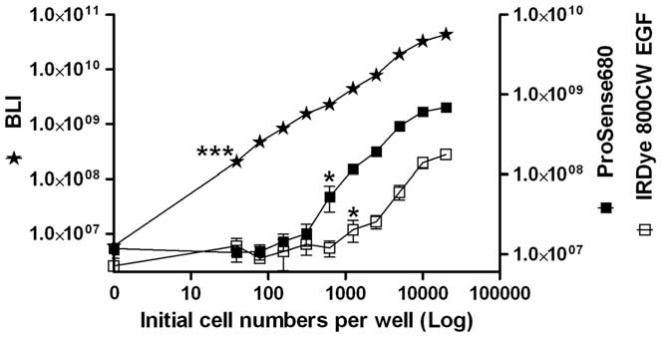
*In vitro* tumor cell detection limit studies. 4T1-luc2 cells were seeded in 96-well plates with various initial densities (39 to 2×10^4^ cells per well). The next day, cells were used for BLI measurement or incubated with either Prosense680 or with IRDye 800CW EGF and then used for FLI measurements. Statistic significance was calculated by determining the difference between optical signals from wells with and without cells. The star indicates the first significant difference. * = P<0.05; *** = P<0.0001.

In order to assess the sensitivity of FLI and BLI measurements, cell limit detection assays were performed. *In vitro*, as shown in Fig. 3, both the bioluminescent and fluorescent signals elevated with increasing cell numbers. However, the sensitivity of BLI was found to be superior to that of FLI. In our study, the cell detection limits were: <39cells/well (P<0.001) for BLI, 625 cells/well (P<0.05) for Prosense680 and 1,250 cells/well (P<0.05) for IRDye 800CW EGF.

**Figure 4:**
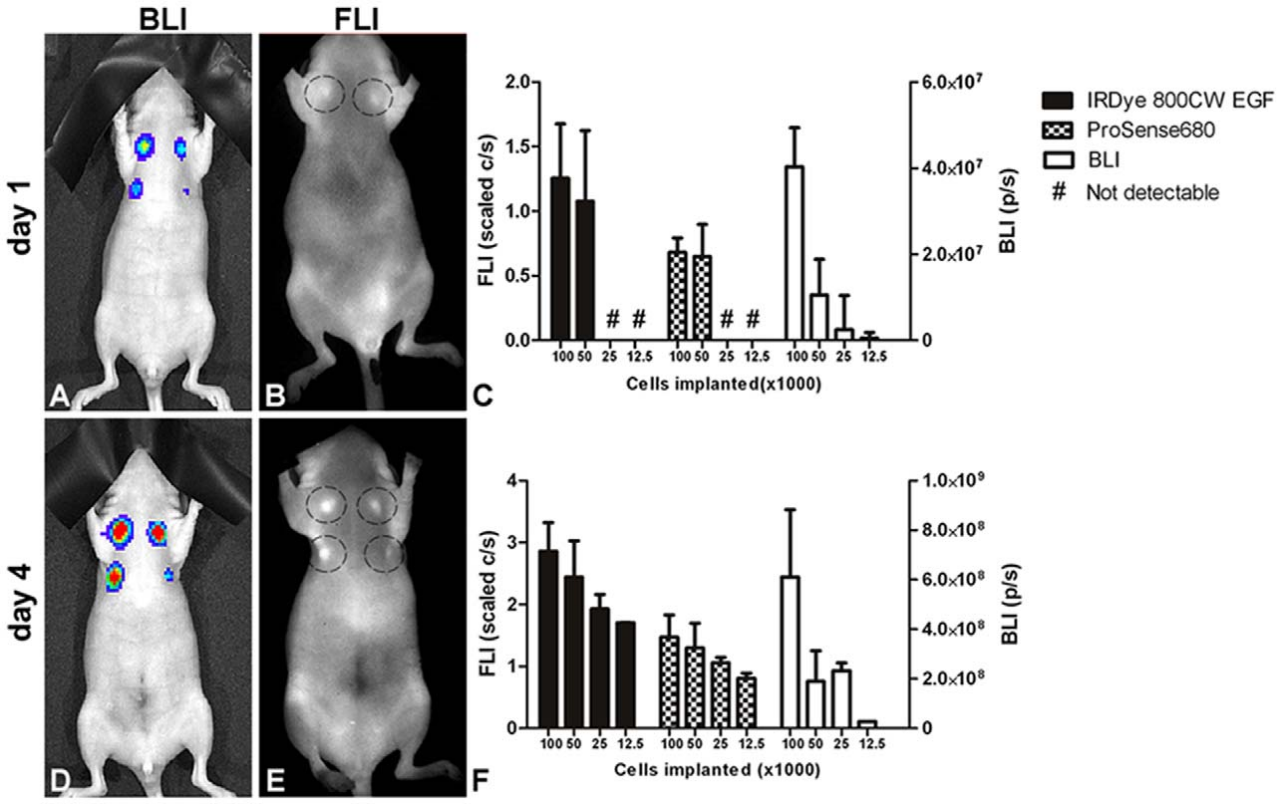
*In vivo* tumor cell detection limit studies. Different numbers of 4T1-luc2 cells were implanted orthotopically into nude mice. 1×10^5^, 5×10^4^, 2.5×10^4^ and 1.25×10^4^ cells were implanted at the upper left, upper right, lower left and lower right MFPs, respectively. BLI measurements at day 1 and 4 are shown in panels A and D. The corresponding IRDye CW800 EGF FLI measurements are shown in panels B and E, respectively. Quantitative analysis of the BLI and FLI measurements using both IRDye CW800 EGF and Prosense680 are shown in panels C and F.

*In vivo*, cell detection limit experiments were performed with orthotopical tumor cell implants. ProSense680 and IRDye 800CW EGF were used for dual-wavelength FLI and compared to BLI measurements. As shown in Fig. 4A and 4C, one day after implantation all MFP implants, including those with the lowest cell number (1.25×10^4^), were detectible with BLI. However, using Prosense680 and IRDye 800CW EGF, the lowest detectible number for FLI was 5×10^4^ cells (Fig. 4B and 4C). Moreover, four days after implantation, all MFP cell implants could be detected both by BLI and FLI (Fig. 4D–F).

### *In vivo* detection of tumor progression

**Figure 5:**
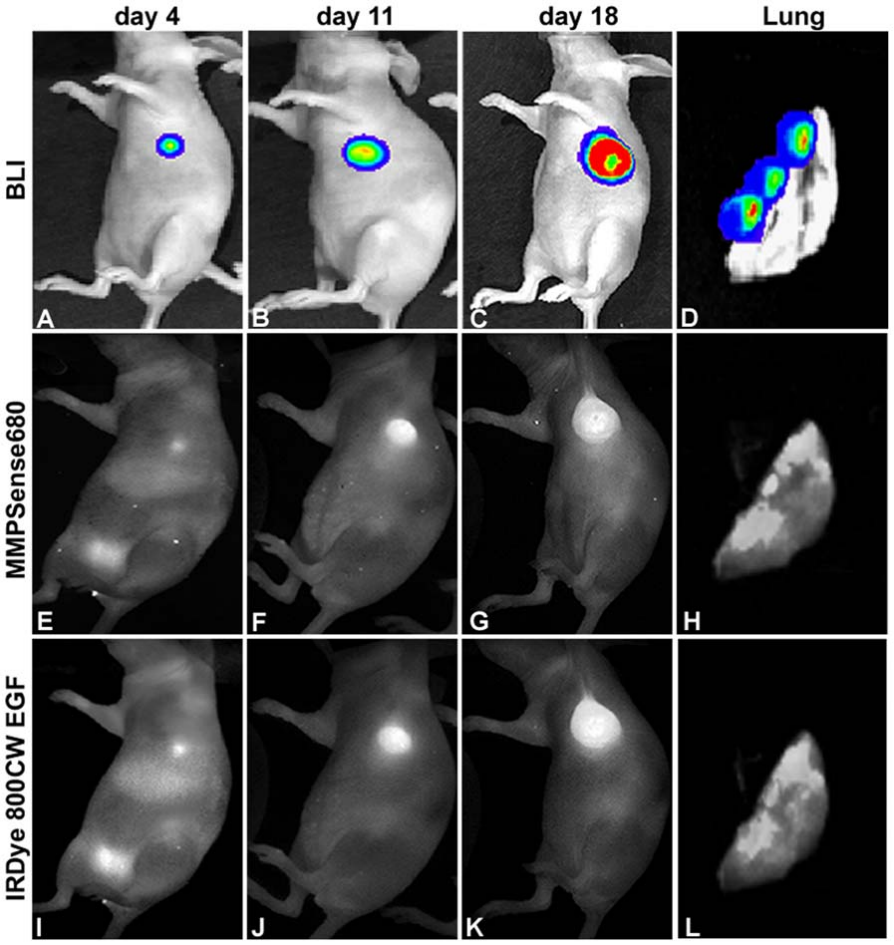
Longitudinal imaging of tumor growth by BLI and FLI. Representative images of tumor progression by BLI (A–D) and FLI utilizing MMPSense680 (E–H) in combination with IRDye 800CW EGF (I–L) are shown. At the end of the experiment, the lungs of the tumor-bearing mice were re-imaged and co-localizing BLI and FLI signals were observed, indicating the presence of pulmonary metastases (D, H and L).

The ability of the four different NIRF probes to detect tumor progression was examined in a mouse model of breast cancer. Both BLI and FLI signals were measured at different time points of tumor progression (day 4 to 18). Dual-wavelength images of MMPSense680 and IRDye 800CW EGF, at different time points of tumor progression, are shown in Fig. 5. As expected, tumors could be detected on day 4 after cell implantation, both by BLI and FLI. The tumor signal intensity increased up to day 18, the final day of the experiment (Fig. 5A–C, E–G and I–K). Some fluorescence was observed in the bladder on day 4 (Fig. 5E and I), due to the excretion of the probes into the urine. This bladder signal was, however, invisible at later time points of tumor progression, as at these stages the bladder signal is superseded by the stronger signal from the tumor. At the end of the experiment, the mice were sacrificed and thoracic cavities were exposed and reimaged to reveal possible metastases. As shown in Fig. 5D, H, L, in the lungs, both BLI and FLI signals were detectible at corresponding sites.

**Figure 6:**
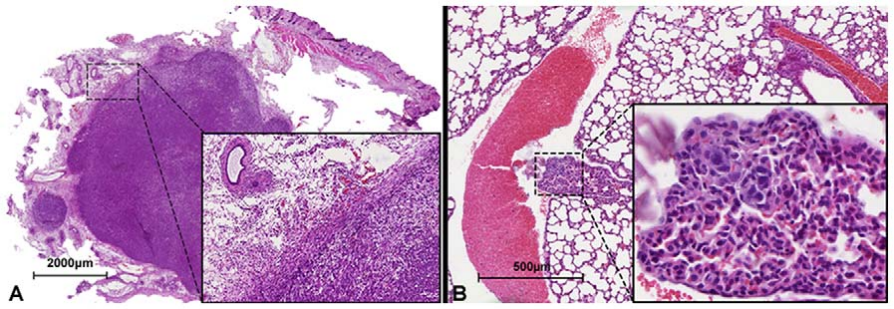
H&E stained paraffin sections of a primary tumor and of its pulmonary metastases. Panel A shows a section of a primary 4T1-luc2 breast tumor indicating neoplastic, actively proliferating cells invading the healthy surrounding tissues. Panel B shows subpleural pulmonary metastases of the tumor.

**Figure 7:**
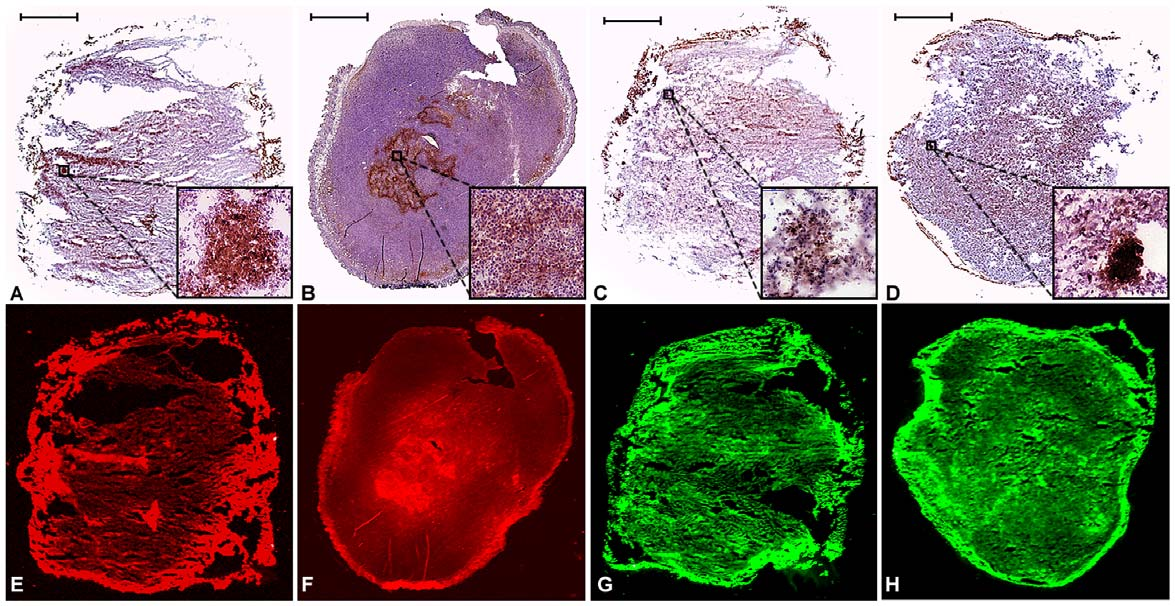
Immunohistological and fluorescent analysis of tumor sections. The upper panels display sections stained with antibodies against EGFR (A), GLUT-1 (B), MMP-9 (C) and CathepsinB (D). The lower panels show the corresponding sections with FLI signals from animals injected with IRDye 800CW EGF (E), IRDye 800CW 2-DG (F), MMPSense680 (G) or ProSense680 (H). Scale bar = 2 mm.

Histological analysis of the primary 4T1-luc2 breast tumor revealed that it composed of actively proliferating neoplastic cells, growing with an incomplete tumor capsule and infiltrating the surrounding tissues (Fig. 6A). The pulmonary metastases, detected by FLI and BLI, were confirmed histologically (Fig. 6B). Moreover, in the tumor sections, the presence of the GLUT-1 and EGFR, as well as the expression of MMP-9 and Cathepsin B, was shown by immunohistochemistry (Fig. 7A–D) as well as indicated by FLI (Fig. 7E–H).

### Correlation between BLI and FLI measurements

**Figure 8:**
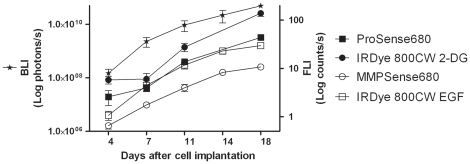
4T1-luc2 tumor growth curve indicated by BLI and FLI. During tumor progression, from day 4 to day 18, there was a steady increase in both BLI and FLI signal intensities.

**Figure 9:**
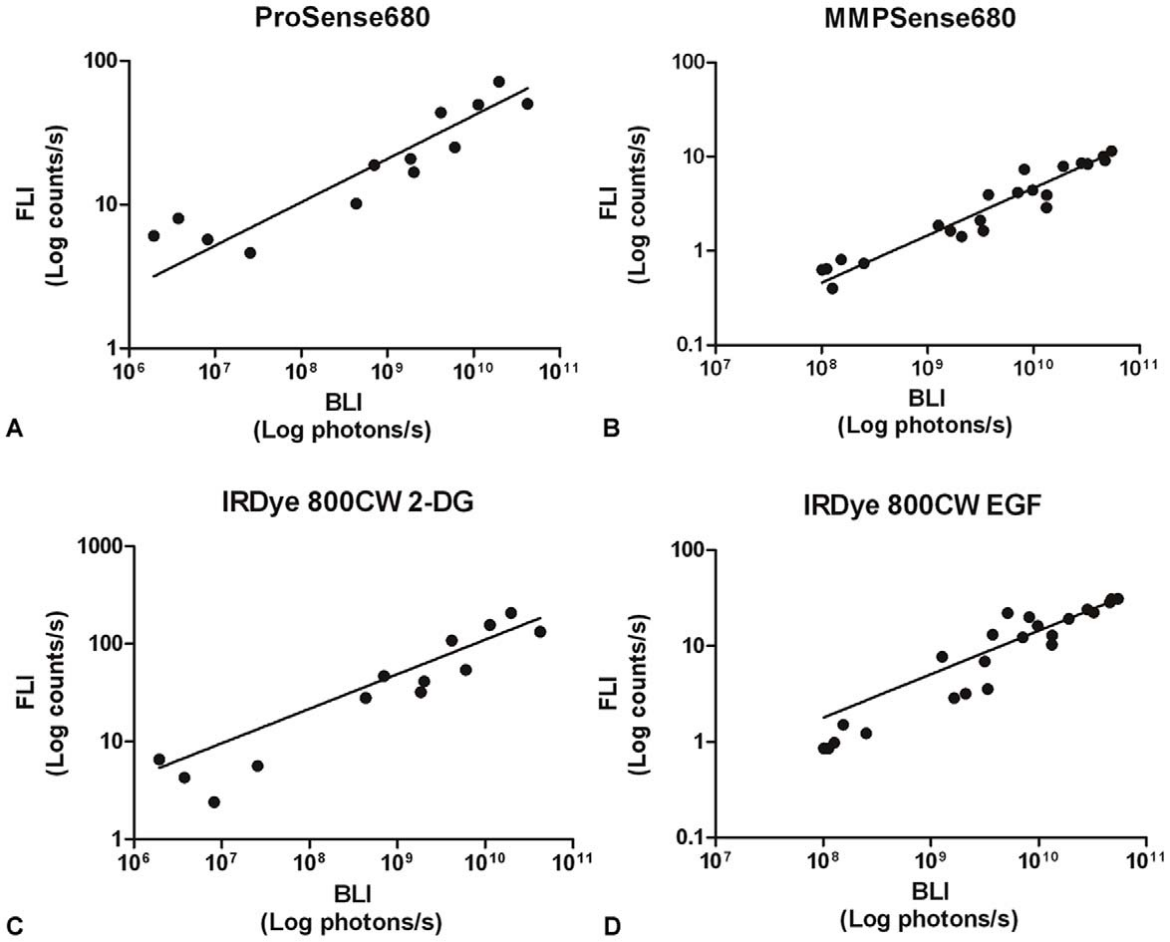
Correlation plots of BLI against FLI signals obtained longitudinally, utilizing four different NIRF probes. The Pearson's r values of the NIRF probes were: Prosense680 (r = 0.72, p<0.01), IRDye 800CW 2-DG (r = 0.70, p<0.01), MMPSense680 (r = 0.92, p<0.0001) and IRDye 800CW EGF (r = 0.88, p<0.0001).

Fig. 8 shows the growth curves of 4T1-luc2 breast tumors detected by BLI and by FLI, using the four different NIRF probes. The correlation plots of the BLI versus FLI signals from the various probes are shown in Fig. 9. A significant linear relationship was found between the obtained tumor BLI and FLI signals from each of the four probes: Prosense680 (r = 0.73, p<0.01), IRDye 800CW 2-DG (r = 0.70, p<0.01), MMPSense680 (r = 0.92, p<0.0001) and IRDye 800CW EGF (r = 0.88, p<0.0001).

### Calculation of the tumor-to-background ratio

**Figure 10:**
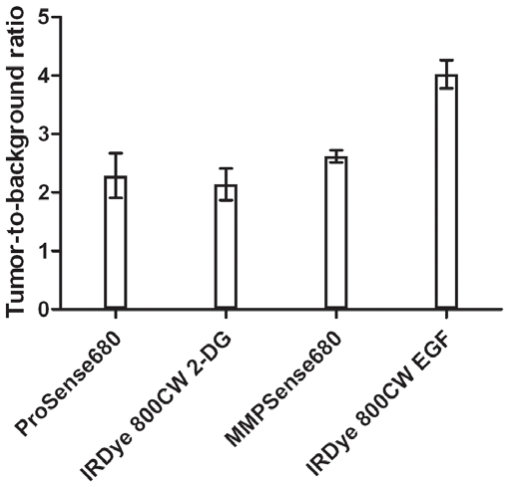
TBRs of 4T1-luc2 tumors exploiting four different NIRF probes. The TBRs, measured on the final day of the experiment, for ProSense680, IRDye 800CW 2-DG, MMPSense680 and IRDye 800CW EGF ranged from 2.29±0.38 to 4.02±0.24.

The calculated TBRs of the different NIRF probes on day 18, the last day of the experiment, were: ProSense680 2.29±0.38; IRDye 800CW 2-DG 2.14±0.27; MMPSense680 2.62±0.1 and IRDye 800CW EGF 4.02±0.24, respectively (Fig. 10).

## Discussion

Given its high sensitivity and negligible background in living tissues, BLI is extensively used for the non-invasive whole body optical imaging of tumor progression in small animals. However, in particular situations, FLI utilizing NIRF injectable probes is a promising alternative to BLI [4], [5], [6], [7], [8]. Moreover, contrary to BLI, FLI can be applied for clinical use. Recently we have successfully implemented FLI in the clinic for sentinel lymph node detection using the NIRF dye indocyanine green (ICG) [33], [34]. In the near future, injectable tumor detecting NIRF probes may enter the clinic, for e.g. image-guided surgery, to facilitate radical tumor resection [9].

To determine if FLI can be used as an alternative for BLI to follow tumor progression, we employed an orthotopic mouse model using the 4T1-luc2 breast cancer cell line. As orthotopically implanted 4T1-luc2 breast cancer cells grow at the primary injection site and can metastasize to various organs within 2–6 weeks, this is an excellent mouse model that closely mimics human breast cancer [29]. Moreover, the introduction of a codon-optimized firefly luciferase (*luc2*) gene into these cells strongly increases the brightness of the bioluminescent signal, allowing for more sensitive and early-stage non-invasive detection of the tumor [2].

In this 4T1-luc2 model, we first examined the tumor detecting abilities of four different NIRF probes, which are able to detect general tumor cell characteristics. These include: increased expression of growth factor receptors (e.g., EGFR) [25], [26], [27], [35], elevated glucose metabolism and up-regulated glucose transporters (e.g., Glut-1) [23], [24] and an increased tissue proteolysis by the tumor, through upregulation of proteolytic enzymes such as MMP-2, -9 [18], [22], [36] and cathepsin B and D [13], [14].

*In vitro*, we examined the activity and binding properties of the four NIRF probes towards 4T1-luc2 cells and observed a dose-dependent uptake of each probe. This indicates that 4T1-luc2 cells express both GLUTs and EGFRs and also possess MMP- and cathepsin- activity *in vitro*. This is in line with previous studies showing that several mammary carcinoma cells, including 4T1 cells, express GLUTs and EGFRs [37], [38], [39] as well as MMP- and cathepsin- activity [40], [41], [42], [43]. Using multispectral microscopy, we demonstrated that the activatable as well as the targeting probes were localized intracellularly. Consistently, Kovar *et al*. previously showed that IRDye CW800 2-DG enters the cytoplasm via internalization of the probe/receptor complex shortly after binding to its receptor [21].

*In vivo*, tumor development and progression was studied using BLI and FLI, exploiting dual-wavelength imaging of a combination of an activatable (∼700nm) and a targeting (∼800 nm) NIRF probe. Tumors could be detected at an early stage of the development (day 4) and a strong linear correlation between FLI and BLI measurements was observed for all probes tested.

Metastases, present in the lungs, could only be detected *ex vivo* both by BLI and FLI and signals co-localized. Histological examination confirmed the presence of subpleural lung metastases. To further examine the specificity of the NIRF probes, immunohistochemistry was performed. Expression of GLUT-1, EGFR, MMP-9 and Cathepsin B was found throughout the tumor and the expression patterns closely corresponded to those obtained with FLI. As can be appreciated from these immunohistological and FLI data, the distribution of the examined probes is rather inhomogeneous in this breast tumor model. The strong immuno-stained areas of each marker coincided with the more intense fluorescent areas. Thus, by using dual-wavelengths, combined with spectral unmixing, it is possible to detect multiple tumor features simultaneously. This may provide a more comprehensive image of the tumor, as different types of tumors are often heterogeneous in structure and molecular characteristics.

The sensitivities of both BLI and FLI *in vitro* and *in vivo* were assessed by tumor cell detection limit experiments. In our study, the *in vitro* detection limit of BLI was lower than 39cells/well. For FLI, the detection limits were 625 and 1,250 cells/well using Prosense680and IRDye 800CW EGF, respectively. *In vivo*, the lowest cell amount (day 1) used for these experiments (1.25×10^4^ cells) was well within the detection limit of BLI, as expected [2]. The detection limits of FLI using IRDye 800CW EGF and Prosense680 were approximately 5×10^4^ cells. Our findings indicate that although the sensitivity of FLI is lower than that of BLI, FLI is a reliable alternative in experimental settings where BLI cannot be used or is not wanted.

The TBR is a ratio which expresses the specificity of a fluorescent probe and as such it is used to discriminate between tumor tissue and healthy tissue. IRDye 800CW EGF had the highest TBR (TBR = 4.02). The TBRs of the other NIRF probes examined in this study were all above two. Probes with a TBR above two can be considered applicable for clinical applications such as image-guided surgery [9], [44].

### Conclusions

FLI using various injectable tumor specific NIRF probes is a feasible approach to follow tumor growth and to detect metastasis. This was shown in sensitivity and specificity studies and by a linear correlation between the FLI and BLI tumor signals. Moreover, dual-wavelength imaging using spectral unmixing enables the simultaneous detection of multiple tumor characteristics. Taken together, dual-wavelength imaging of tumor progression by activatable and targeting NIRF probes is useful in pre-clinical studies and can possibly be translated towards clinical applications, such as image-guided surgery.
